# Association of Endotracheal Aspirate Culture Variability and Antibiotic Use in Mechanically Ventilated Pediatric Patients

**DOI:** 10.1001/jamanetworkopen.2021.40378

**Published:** 2021-12-22

**Authors:** Andrea Prinzi, Sarah K. Parker, Cary Thurm, Meghan Birkholz, Anna Sick-Samuels

**Affiliations:** 1 Department of Infectious Diseases, Children’s Hospital Colorado, Denver; 2 University of Colorado Anschutz Medical Campus Graduate School, Denver; 3 Department of Pediatrics, University of Colorado School of Medicine, Denver; 4 Children’s Hospital Association, Lenexa, Kansas; 5 Department of Pediatrics, Johns Hopkins University School of Medicine, Baltimore, Maryland

## Abstract

**Importance:**

Endotracheal aspirate cultures are commonly collected from patients with mechanical ventilation to evaluate for ventilator-associated pneumonia or tracheitis. However, the respiratory tract is not sterile, making differentiating between colonization from bacterial infection challenging, and results may be unreliable owing to variable specimen quality and sample processing across laboratories. Despite these limitations, clinicians routinely interpret bacterial growth in endotracheal aspirate cultures as evidence of infection, sometimes regardless of organism significance, prompting antibiotic treatment.

**Objective:**

To assess the variability in endotracheal aspirate culture rates and the association between culture rates and antibiotic prescribing among patients with mechanical ventilation across children’s hospitals in the US.

**Design, Setting, and Participants:**

Cross-sectional retrospective analysis of data obtained from the Children’s Hospital Association Pediatric Health Information System database between January 1, 2016, through December 31, 2019. Participants were all patients hospitalized with mechanical ventilation aged less than 18 years.

**Exposures:**

A charge for an endotracheal aspirate culture on a ventilated day.

**Main Outcomes and Measures:**

Endotracheal aspirate culture rate and antibiotic days of therapy per ventilated days. For mechanical ventilation, clinical transaction classification codes for mechanical ventilation other unspecified ventilator assistance were used. To identify respiratory cultures, the laboratory test code for aerobic culture was used and relevant keywords (ie, respiratory tract, sputum) were used to identify sources in the hospital charge description master.

**Results:**

A total of 152 132 patients were identified among 31 hospitals. Among these patients, 79 691 endotracheal aspirate cultures were collected on a ventilator-day (patients aged less than 1 year, 44%; 1-4 years, 27%, 5-11 years. 16%, and 12-18 years, 13%; 3% were Asian; 17% Hispanic; 21% non-Hispanic Black; 45% Non-Hispanic White patients; 14% were other; 56% of patients were male, 44% were female). The overall median rate of culture use was 46 per 1000 ventilator-days (IQR, 32-73 cultures per 1000 ventilator-days). The endotracheal aspirate culture rate was positively correlated with the hospital’s antibiotic days of therapy rate (*R* = 0.46; *P* = .009). In a multivariable model adjusting for patient-level and hospital-level characteristics and among patients with mechanical ventilation, each additional endotracheal aspirate culture was associated with 2.87 (95% CI, 2.74-3.01) higher odds of receiving additional days of therapy compared with patients who did not receive and endotracheal aspirate culture.

**Conclusions and Relevance:**

In this study, notable variability was found in endotracheal aspirate culture rates across US pediatric hospitals and pediatric intensive care units, and endotracheal aspirate culture use was associated with increased antibiotic use. These findings suggest an opportunity for diagnostic and antibiotic stewardship to standardize testing and treatment of suspected ventilator-associated infections in pediatric patients with mechanical ventilation pediatric patients.

## Key Points

**Question:**

Is endotracheal aspirate culture (EAC) use variable across the US, and is EAC use associated with antibiotic use in children receiving mechanical ventilation?

**Findings:**

In this cross-sectional study of 31 pediatric hospitals and 152 132 patients in the US, EAC use varied widely between hospitals, and higher rates were correlated with increased antibiotic use. Patients who underwent an EAC culture were significantly more likely to receive antibiotics after adjustment for patient demographic characteristics, patient complexity, complex conditions, duration, and episodes of mechanical ventilation.

**Meaning:**

In this study, EACs are associated with increased antibiotic prescribing in hospitalized mechanically ventilated children may represent an opportunity for diagnostic and antibiotic stewardship.

## Teaser

This cross-sectional study characterizes the variability in the rate of endotracheal aspirate culture use and assesses the association between endotracheal aspirate culture use and antibiotic use across US pediatric hospitals.

## Introduction

Ventilator-associated pneumonia (VAP) is the most frequently acquired infection in patients with mechanical ventilation and the second most common nosocomial infection in the intensive care unit (ICU).^1^ In addition to increased length of stay, VAP is associated with an extended duration of ventilation, increased risk of morbidity and mortality, and high costs.^2,3^ There is no standard definition for VAP, which makes the diagnosis challenging. Therefore, some clinicians may have a low threshold to obtain endotracheal aspirate cultures (EACs) to evaluate for suspected VAP or VAT.^1,4,5^

The results from EACs are relatively nonspecific for infection and may be unreliable.^6,7,8,9,10,11,12,13,14,15,16^ Sample collection and quality of specimens are variable, which may affect results.^16,17^ Endotracheal secretion samples are from a nonsterile site, making the differentiation between colonization and infection in the culture challenging. There is also a substantial amount of variability in the way EACs are processed and reported,^18^ which further complicates result interpretation and may be a factor in EAC unreliability. Clinicians may misinterpret reported growth of bacteria in EAC results as being an indicator of infection and then treat with antibiotics.^1,19,20^ Therefore, overuse of EACs may potentiate avoidable antibiotic treatment in patients with mechanical ventilation and contribute to avoidable harms such as *Clostridioides difficile* infections, antimicrobial resistance,^21^ and adverse drug reactions.^22,23^ Ultimately, EAC practices may vary widely within and across institutions. We aimed to characterize the variability in the rate of EAC use and assess the association between EAC use and antibiotic use across US pediatric hospitals.

## Methods

This study was a retrospective multicenter cross-sectional analysis of clinical and billing data from the Pediatric Health Information System (PHIS, Children’s Hospital Association, Overland Park, Kansas) from January 1, 2016, through December 31, 2019. PHIS is a national database that compiles administrative data including clinical and resource use from more than 49 children’s hospitals geographically dispersed across the US. Hospitals that did not continuously submit billing data to PHIS during the study period were excluded (n = 18). Data were obtained for all inpatient units and further stratified into ICU and neonatal ICU (NICU) groups for analysis. Of note, the ICU subgroup contained data from pediatric ICUs (PICUs) and pediatric cardiac ICUs because division of these units varies by hospital and is not easily distinguished in the PHIS database. This study was classified as exempt research (secondary research not requiring informed patient consent) per the University of Colorado institutional review board. This study followed the Strengthening the Reporting of Observational Studies in Epidemiology (STROBE) reporting guideline for cross-sectional studies.

### Patient Population

This study included all patients less than age 18 years who were hospitalized at a participating PHIS hospital during the study period and had at least 1 day of mechanical ventilation. We identified patients with mechanical ventilation as those with daily Clinical Transaction Codes (CTCs) for mechanical ventilation (n = 152 132), which includes all types of invasive ventilation (ie, tracheostomy, endotracheal, and nasotracheal). The CTC code is associated with a day of service each time it is used and allows for the calculation of ventilator-days for each patient. Patients who only had an *International Statistical Classification of Diseases and Related Health Problems, Tenth Revision (ICD-10)* procedure code for ventilation without mechanical ventilation CTC codes and patients with missing ventilation coding data were excluded (n = 4546) because the duration of ventilation could not be ascertained. To assess the accuracy of mechanical ventilation billing codes, we performed a validation using local Children’s Hospital Colorado data. This validation demonstrated that the use of *ICD-10* codes for intubation and ventilation excluded data on patients with tracheostomy and made duration of ventilation undeterminable; they were therefore excluded.

### Primary Outcomes

For this study, we defined the rate of EAC use as the total number of EACs billed for on a ventilated day per 1000 total ventilator-days. We identified EACs when the patient had a code for respiratory aerobic culture coinciding with a charge for ventilation on the same calendar day. Specimen source billing data were reviewed for consistency by a microbiologist (A.P.) and infectious disease physician (A.S.) and refereed by an additional infectious disease physician (S.K.P.). Because laboratory test descriptions and specimen source descriptions may be nonspecific (ie, aerobic culture, other specimen source), associated hospital charge description data were reviewed for details that specified the specimen source (eTable 1 in the Supplement). All respiratory tract, nasopharyngeal, sputum, other, and unspecified sources with charge descriptions that included descriptors such as bacterial culture and tracheal aspirate from a patient receiving mechanical ventilation were considered to be an EAC specimen. We excluded bronchoalveolar lavage specimens, nasopharyngeal specimens used for methicillin-resistant *Staphylococcus aureus* screening, viral respiratory testing, anaerobic culture, and any additional testing unrelated to standard aerobic bacterial culture such as *Pneumocystis* direct fluorescent antibody and fungal testing. Rates of use were calculated for all inpatient units, the ICU subgroup, and the NICU subgroup.

We defined the rate of antibiotic use as an antibiotic day of therapy (DOT) occurring on a ventilated day per 1000 ventilator-days. An antibiotic DOT was identified when the patient had a billing code for an antibiotic coinciding with a charge for ventilation on the same calendar day. We included a comprehensive list of antibiotics given via enteral or intravenous methods commonly prescribed for ventilator-associated infections (VAIs) (eTable 2 in the Supplement). We excluded rarely used antibiotics and inhaled antibiotics (eTable 3 in the Supplement). We further subanalyzed anti-*Pseudomonal* and anti-*Staphylococcal* antibiotics because *Pseudomonal* and *Staphylococcal* organisms are commonly recovered from EACs and are associated with chronic colonization.^24,25^ Additionally, we considered a subanalysis of antibiotics for highly drug-resistant organisms (eg, ceftazidime-avibactam); however, there was an insufficient volume of DOT. Rates of use were calculated for all inpatient units, the ICU subgroup, and the NICU subgroup.

### Patient and Hospital-Level Characteristics

Characteristics associated with EAC use and antibiotic use were considered a priori and informed by previously reported associations conducted using the PHIS database.^26^ Variables collected for each patient for each hospital admission included age (<1 year, 1-4 years, 5-11 years, and 12-18 years), race and ethnicity (Asian, non-Hispanic Black, non-Hispanic White, other), sex, the presence of any chronic condition, the patient’s cumulative number of ventilator-days and their number of ventilator episodes during the admission. Variables characterizing the hospital included case-mix index^27^ and geographic location (Midwest, Northeast, South, West). Patient race and ethnicity data were collected at each individual hospital at the time of patient registration, and were not determined by the investigators.^28^

### Statistical Analysis

Characteristics were summarized using frequencies and percentages for categorical variables, and their association with EAC or antibiotic use was first compared using χ^2^ tests. The Shapiro-Wilk test was used to evaluate the distribution of each variable, and Pearson (normal) and Spearman (nonnormal) correlation tests were used accordingly to assess the correlation between EAC use and antibiotic use. All correlation analyses were performed using R studio, version 1.4.1106 (R Studio). Covariates that were significantly associated with receiving an antibiotic on a ventilated day were entered into the final model for adjustment to accurately assess the association between EAC and antibiotic use in patients with mechanical ventilation patients.

Adjusted EAC use and antibiotic DOT rates per 1000 total ventilator-days were calculated using generalized linear mixed-effects models with Poisson distributions, controlling for hospital-level clustering and allowing for the presence of correlated data (within hospital), nonconstant variability (across hospitals), and responses that are not normally distributed. We used the same approach to assess DOT per 1000 total ventilator-day values for all patients, patients in the ICU, patients in the NICU across institutions, and the antibiotic groups (all antibiotics, anti-*Pseudomonal,* and anti-*Staphylococcal*). Covariates that had a significant univariate association with antibiotic use and determined a priori to be clinically relevant were included in the final model. Of note, the association between number of chronic conditions as well as individual chronic conditions and EAC use was assessed (eTable 4 in the Supplement) and had significant collinearity. Due to this collinearity, a binary variable of any complex chronic condition was instead included in the final model. Statistical significance for covariate selection and the main model was based on a 2-sided analysis and a *P* value less than .01 because of the large sample size. Analyses were performed using SAS software, version.9.4 (SAS Institute).

## Results

### EAC Use

**Figure 1.**
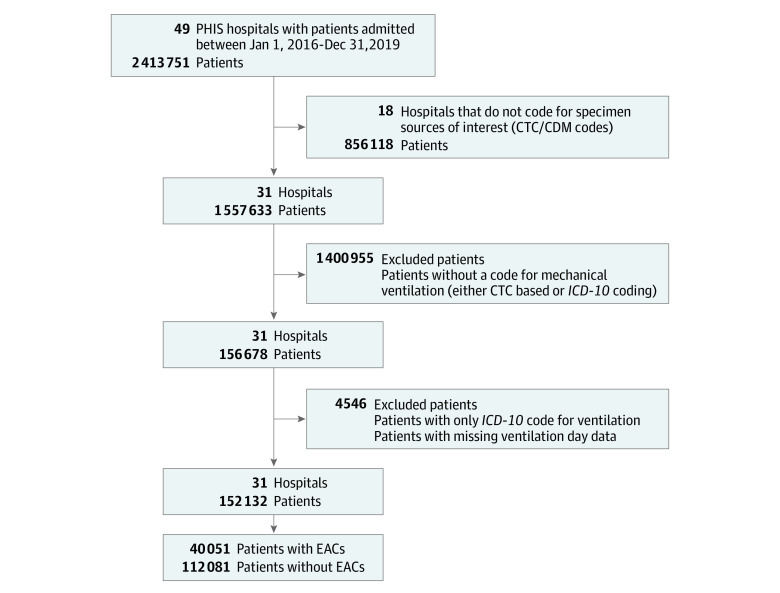
Flowchart of Identification of Patients With Mechanical Ventilation and Hospitalization CTC/CDM indicates Clinical Transaction Codes/cut-down method; EACs, endotracheal aspirate cultures; *ICD-10*, *International Statistical Classification of Diseases and Related Health Problems, Tenth Revision*; PHIS, Pediatric Health Information System.

Of the 51 PHIS hospitals, 31 hospitals met inclusion criteria, and 152 132 patients were admitted and received mechanical ventilation on a ventilated day during the study period (Figure 1). Among these patients, 79 691 EAC were collected on a ventilator day. Most patients in the study cohort were less than 1 year of age (44%) followed by 1-4 years (27%), 5-11 years (16%) and 12-18 years (13%). For race and ethnicity, 3% were Asian; 17% Hispanic; 21% non-Hispanic Black; 45% Non-Hispanic White patients; 14% were other. With respect to sex, 56% of patients were male, 44% were female. The Table describes demographic characteristics, culture, and antibiotic use for all included hospitals.

**Figure 2.**
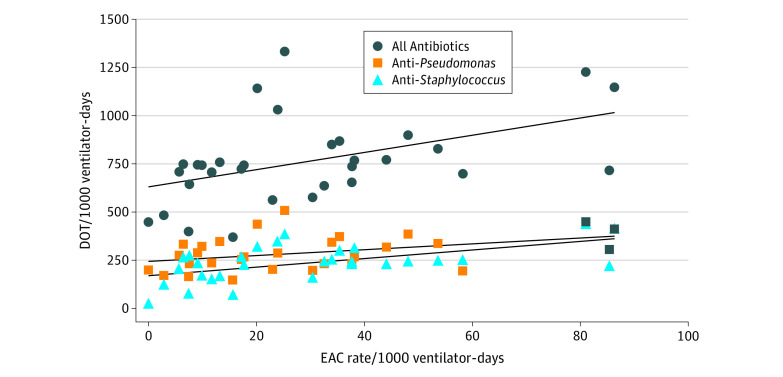
EAC Rate vs Days of Therapy, All Units (2016-2019) DOT indicates day of therapy; EAC, endotracheal aspirate culture.

Overall, the unadjusted median rate of EAC use for all units and all hospitals was 46 per 1000 total ventilator-days (IQR, 32-73 cultures per 1000 total ventilator-days) (Figure 2). EAC use for the ICU group was 100 per 1000 total ventilator-days (IQR, 63-137 per 1000 total ventilator-days) and for the NICU group was 24 per 1000 total ventilator-days (IQR, 11-34 per 1000 total ventilator-days). Before adjustment, numerous variables were significantly associated with receiving an EAC test, including case mix index (OR, 1.03 [95% CI, 1.02-1.03]; *P* < .001), non-Hispanic Black race (OR, 1.16 [95% CI, 1.13-1.20]; *P* < .001), and presence of any chronic condition (OR, 2.13 [95% CI, 2.06-2.20]; *P* < .001) (Table).

**Table.** Association of Patient and Hospital-Level Characteristics With Receiving an Endotracheal Aspirate Culture (EAC) and Antibiotic DOT

Variable	Patients with mechanical ventilation, No. (%)		Odds ratio (95% CI) of receiving EAC		Odds ratio (95% CI) of receiving antibiotics	
	Without EAC (n = 112 081)	With EAC (n = 40 051)	Unadjusted	Adjusted	Unadjusted	Adjusted
Case-mix index, median (IQR)	6.34 (2.91-14.36)	8.05 (3.89-16.45)	1.03 (1.02-1.03)^a^	1.03 (1.02-1.03)^a^	1.17 (1.17-1.17)^a^	1.11 (1.11-1.12)^a^
Geographic location						
Midwest	37 039 (33)	12 539 (31.3)	0.67 (0.65-0.69)^a^	0.83 (0.42-1.62)	0.58 (0.56-0.60)^a^	1.01 (0.73-1.38)
Northeast	24 269 (21.7)	5875 (14.7)	0.48 (0.46-0.50)^a^	0.42 (0.19-0.91)	0.56 (0.54-0.59)^a^	0.83 (0.57-1.20)
South	33 492 (29.9)	12 908 (32.2)	0.76 (0.74-0.79)^a^	0.81 (0.42-1.56)	1.02 (0.98-1.07)	1.11 (0.81-1.51)
West	17 281 (15.4)	8729 (21.8)	1 [Reference]	1 [Reference]	1 [Reference]	1 [Reference]
Age, y						
<1	65 256 (58.2)	17 450 (43.6)	0.63 (0.61-0.65)^a^	0.61 (0.59-0.64)^a^	1.54 (1.48-1.60)^a^	1.27 (1.21-1.33)^a^
1-4	20 228 (18)	10 761 (26.9)	1.25 (1.20-1.30)^a^	1.31 (1.25-1.36)^a^	1.05 (1.01-1.10)^a^	1.09 (1.04-1.14)^a^
5-11	14 087 (12.6)	6518 (16.3)	1.09 (1.04-1.14)^a^	1.05 (1.00-1.10)	1.24 (1.18-1.30)^a^	1.25 (1.18-1.32)^a^
12-18	12 510 (11.2)	5322 (13.3)	1 [Reference]	1 [Reference]	1 [Reference]	1 [Reference]
Sex						
Male	63 336 (56.5)	22 414 (56)	0.98 (0.96-1.00)	1.01 (0.98-1.03)	1.00 (0.97-1.02)	1.01 (0.98-1.04)
Female	48 745 (43.5)	17 637 (44)	1 [Reference]	1 [Reference]	1 [Reference]	
Race and ethnicity						
Asian	3789 (3.4)	1167 (2.9)	0.92 (0.86-0.99)	1.04 (0.97-1.12)	1.07 (0.99-1.15)	0.95 (0.87-1.03)
Hispanic	17 176 (15.3)	6919 (17.3)	1.21 (1.17-1.24)^a^	1.08 (1.04-1.12)^a^	1.34 (1.29-1.40)^a^	0.98 (0.94-1.03)
Non-Hispanic Black	21 688 (19.4)	8446 (21.1)	1.16 (1.13-1.20)^a^	1.20 (1.16-1.24)^a^	0.87 (0.84-0.90)^a^	0.80 (0.77-0.83)^a^
Non-Hispanic White	53 575 (47.8)	17 910 (44.7)	1 [Reference]	1 [Reference]	1 [Reference]	1 [Reference]
Other^b^	15 853 (14.1)	5609 (14)	1.06 (1.02-1.10)^a^	1.09 (1.05-1.14)^a^	1.03 (0.99-1.07)	0.98 (0.94-1.03)
Any CCC	84 029 (75)	34 626 (86.5)	2.13 (2.06-2.20)^a^	0.95 (0.91-0.99)^a^	3.66 (3.56-3.76)^a^	1.55 (1.50-1.60)^a^
Cumulative ventilator-days						
1-3	72 351 (64.6)	7993 (20)	1 [Reference]		1 [Reference]	1 [Reference]
4-14	31 176 (27.8)	18 763 (46.8)	5.45 (5.29-5.61)^a^	2.60 (2.50-2.71)^a^	3.80 (3.68-3.93)^a^	1.88 (1.81-1.95)^a^
>14	8554 (7.6)	13 295 (33.2)	14.07 (13.58-14.58)^a^	6.81 (6.40-7.24)^a^	11.01 (10.25-11.83)^a^	1.87 (1.72-2.03)^a^
No. of ventilation episodes						
1	95 594 (85.3)	28 662 (71.6)	1 [Reference]	1 [Reference]	1 [Reference]	1 [Reference]
≥2	16 487 (14.7)	11 389 (28.4)	2.30 (2.24-2.37)^a^	0.86 (0.83-0.90)^a^	3.81 (3.63-3.99)^a^	1.25 (1.18-1.32)^a^
Receiving EAC						
No	NA	NA	NA	NA	1 [Reference]	1 [Reference]
Yes	NA	NA	NA	NA	4.68 (4.49-4.88)^a^	2.87 (2.74-3.01)^a^

Abbreviations: CCC, complex chronic condition; EAC, endotracheal aspirate; NA, not applicable.

^a^ Statistical significance defined as *P < *.001.

^b^ Including American Indian, Alaskan Native, Native Hawaiian Pacific Islander, and other meaning all other race and ethnicity groups including those records that have multiple race groups indicated on their patient record.

**Figure 3.**
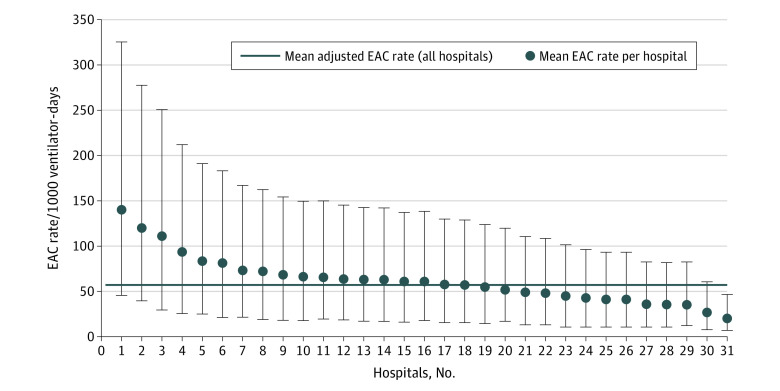
Adjusted EAC Rate per 1000 Ventilator-Days The whiskers represent 95% CIs. EAC indicates endotracheal aspirate culture.

After adjustment, the median rate of EAC for all hospitals and units was 57 per 1000 total ventilator-days. The adjusted EAC rates for individual institutions ranged from 20 per 1000 total ventilator-days (95% CI, 13-26) to 119 per 1000 total ventilator-days (95% CI, 80-158) (Figure 3). Case-mix index was significantly associated with receiving an EAC (adjusted odds ratio [AOR], 1.03 [95% CI, 1.02-1.03]), and the odds of receiving EAC testing was highest in the 1–4-year age group (AOR, 1.31 [95% CI, 1.25-1.36]) (Table). The odds of EAC testing were significantly higher in non-Hispanic Black patients compared with non-Hispanic White patients (AOR, 1.08 [95% CI, 1.04-1.12]). The variables that were associated with EAC use in the final model included the presence of underlying chronic conditions (AOR, 0.95 [95% CI, 0.91-0.99]; *P* < .001), the number of cumulative ventilator-days a patient had (>14 days, AOR, 2.60 [95% CI, 2.50-2.71]; *P* < .001), and the total number of ventilation episodes (2 or more, AOR, 0.86 [95% CI, 0.83-0.90]; *P* < .001) (Table).

### Antibiotic Use

For all antibiotics, the unadjusted median DOT rate was 783 DOT per 1000 total ventilator-days across all units (IQR, 686-875 per 1000 total ventilator-days), 1181 DOT per 1000 total ventilator-days in the ICU (IQR, 1062-1387 per 1000 total ventilator-days), and 740 per 1000 total ventilator-days in the NICU (IQR, 648-838 per 1000 total ventilator-days). Before adjustment, numerous variables were associated with antibiotic use including age (<1 year, OR, 1.54 [95% CI, 1.48-1.60]; *P* < .001), race (non-Hispanic Black, OR, 0.87 [95% CI, 0.84-0.90]; *P* < .001) and cumulative ventilator days (4-14 days, OR, 3.80 [95% CI, 3.68-3.93]; *P* < .001) (Table).

 Except for hospital geographic location, all patient and hospital characteristics that were significantly associated with receiving an antibiotic on a ventilator day were also significantly associated after adjustment. Non-Hispanic Black patients had significantly less odds of receiving antibiotics on a ventilated day than non-Hispanic White patients (AOR, 0.80 [95% CI, 0.77-0.83]). Additionally, the odds of receiving an antibiotic was 1.55 (95% CI, 1.50-1.60) times higher in patients with at least 1 chronic condition compared with patients without and odds of antibiotic use were higher in patients with more cumulative ventilator days (4-14 days, AOR 1.88 [95% CI, 1.81-1.95]).

Across all units, there was a positive correlation between EAC rate and DOT (*R* = 0.46; *P* = .009). In the ICU subgroup, the EAC rate was positively correlated with the DOT for all antibiotics (*R* = 0.48; *P* = .001) and anti-*Staphylococcal* drugs (*R* = 0.43; *P* = .015). However, in this ICU subgroup, the EAC rate was not correlated with anti-*Pseudomonal* antibiotics (*R* = 0.28; *P* = .12). In the NICU subgroup, the EAC rate had a positive correlation with antibiotic DOT for all antibiotics (*R* = 0.5; *P* = .005), anti-*Pseudomonal* antibiotics (*R* = 0.39; *P* = .03), and anti-*Staphylococcal* antibiotics (*R* = 0.51, *P* = .004).

After accounting for significant patient and hospital characteristics in the final model, patients who received EAC testing on a ventilated day had 3 times the odds of receiving an antibiotic than patients with mechanical ventilation who did not receive EAC testing (AOR, 2.87 [95% CI, 2.74-3.01]). Patients with mechanical ventilation with EACs were also more likely to receive anti-*Pseudomonal* antibiotics (AOR, 2.01 [95% CI, 1.95-2.07]) and anti-*Staphylococcal* antibiotics (AOR, 2.04, [95% CI, 1.97-2.10]) compared with patients with mechanical ventilation without EACs.

## Discussion

In this observational study of EAC use in 31 pediatric hospitals from 2016 to 2019, we found variability in the rate of EAC use. Some patient-level characteristics were associated with higher EAC use such as age, presence of a complex chronic condition, cumulative ventilator-days, and the number of ventilation episodes, which aligns with clinical expectations. Some of the associations were not anticipated, such as non-Hispanic Black patients having a higher rate of EAC use but lower antibiotic use than White patients. More investigation is warranted for a better understanding of these observed differences in testing and treatment by race, which has been seen in other diagnostic evaluations.^29^ After adjustment for patient and hospital characteristics, there was still a 10-fold difference in the highest and lowest hospital-level rate of EAC use, suggesting that differences in patient populations do not fully explain variability in testing frequency.

When examining the association between EAC and antibiotic usage, we found a moderate positive correlation. The multivariable modeling also showed increased odds of antimicrobial use in patients with mechanical ventilation who received more EAC testing. Overall, the antibiotic rates in this population with mechanical ventilation and associated variables align with prior studies^26,30^ in pediatric populations. We aimed to explore whether trends could be found when focusing on anti-*Pseudomonal* or anti-*Staphylococcal* antibiotics because these organisms can cause chronic colonization and biofilm formation of airway devices.^31^ A strong correlation was not found for anti-*Pseudomonal* agents except in the NICU subgroup. Although this study included antibiotics prescribed only on ventilated days and medications commonly used to treat VAIs, billing data cannot provide the level of granularity to determine specific treatment indications. Particularly in the PICU setting, anti-*Pseudomonal* agents such as cefepime are commonly used for other infectious processes, so a portion of the captured antibiotic treatment may possibly be unrelated to VAI management.

Overall, one may expect that culture use is associated with antibiotic prescribing rates because clinicians will treat for suspected infections. The observational ecologic (ie, population-level) design of this study cannot determine directionality or causality between EAC testing frequency and antibiotic usage. Specifically, we did not have the data to independently adjudicate appropriateness of individual EACs or specific antibiotic treatment indications. However, prior studies suggest that EAC testing may be associated with additional antibiotic treatment.^6,21,32,33,34,35^ For example, in the first of two ventilator-associated infection studies (VAIN),^36^ a multicenter study across 22 PICUs, antibiotic treatment was associated with the patient having had a positive EAC, and in the second VAIN study, implementing guidelines to de-escalate antibiotic treatment if the patient did not exhibit signs of respiratory infection was only successful in 27% of cases, largely owing to clinicians continuing treatment for positive EACs.^37^ Furthermore, recent reports from pediatric hospitals implementing clinical decision support for EAC testing were associated with significant reductions in EAC use and antibiotic prescribing for indication of VAIs.^38,39^ Therefore, we believe that in the present study, at least a portion of the EAC testing among hospitals with higher EAC use may be associated with higher antibiotic usage.

A complicating feature of EACs is that the respiratory secretions are sampling a nonsterile body site; therefore EACs may be limited in their ability to differentiate between bacteria colonizing the airway from those acting as pathogens.^16,19,40,41,42^ Multiple studies have shown no association between bacterial growth or inflammation with clinical findings of respiratory infection across pediatric and adult populations.^16,19,41,43^ Various factors unrelated to the culture may affect the likelihood of infection such as inflammation, clinical status of the patient, and environmental exposures.^25,44^ Recognizing that VAI may have substantial morbidity if untreated, reporting of bacteria in EAC results presents a challenge for the clinician and may encourage antibiotic use regardless of the clinical relevance of the organism.^24^ The Infectious Disease Society of America recognized that EACs were less specific for infection and associated with more antibiotic use compared with bronchoalveolar lavage (BAL) specimens, but because of less patient risk and cost, the VAP guidelines recommended EACs over BAL cultures.^45^ Compounding these known limitations of EACs, variability occurs in sample quality, processing and reporting of EACs across US microbiology laboratories.^18^ Diagnostic stewardship interventions and formal guidelines for collecting, processing, and reporting for EACs are needed to support accurate interpretation and antibiotic treatment decisions for suspected VAI.

Clinician variability in pediatric VAI management practices has been described^5,46^ and the present study further confirms interhospital variability in EAC testing practices. These descriptions are not isolated to pediatric care, lack of standardization, potential harms of EAC overuse, and diagnostic stewardship interventions have been proposed for adult patients with mechanical ventilation.^47,48,49^ Additional study is warranted to examine the association between EAC testing patterns and antibiotic use across the ages spectrum, emergency and ambulatory care settings, while also considering clinician and institutional factors.

### Limitations

This study has limitations. A primary limitation of this study is the use of billing data, which can lead to misclassification. The use of ventilator-days to identify patients did not capture patients or EACs from tracheostomies if the patient was not ventilated. If an EAC was charged under a different billing code, it could have been omitted or some EACs may have been mislabeled and in fact been a different specimen. To optimize data validity, we excluded hospitals with inconsistent data reporting. Additionally, to ensure patients with mechanical ventilation were correctly identified, we back-validated PHIS data with Children’s Hospital Colorado patient data. To reduce the risk of misclassification of specimen type, we reviewed details of hospital charge descriptions. Although we captured important hospital and patient-level characteristics, billing data cannot reflect all the potentially relevant patient clinical condition details or local environment such as illness severity, indications for testing and treatment, the role of antibiotic stewardship programs, or clinician experience that may be associated with EAC and antibiotic usage. In particular, we could not distinguish the type of artificial airway (tracheostomy vs endotracheal tube).

## Conclusions

The results from this cross-sectional study found notable variability in EAC rates of use across pediatric hospitals, and EAC use was associated with higher odds of antibiotic use. Given the diagnostic limitations of EACs, these findings suggest that there may be opportunity for diagnostic and antibiotics stewardship to standardize EAC testing and treatment among children with mechanical ventilation.

## Notes

Supplement. **eTable 1.** Billing Codes **eTable 2.** Included Antibiotics **eTable 3.** Excluded Antibiotics **eTable 4.** Association Between Complex Chronic Conditions and Receiving EAC Testing
