# Supplementary material for: Association of Endotracheal Aspirate Culture Variability and Antibiotic Use in Mechanically Ventilated Pediatric Patients
Source: JAMA Netw Open. 2021 Dec 22;4(12):e2140378. doi: 10.1001/jamanetworkopen.2021.40378 (PMC8696566; doi:10.1001/jamanetworkopen.2021.40378)
Supplement: Supplement. — eTable 1. Billing Codes eTable 2. Included Antibiotics eTable 3. Excluded Antibiotics eTable 4. Association Between Complex Chronic Conditions and Receiving EAC Testing [file jamanetwopen-e2140378-s001.pdf]

Supplemental Online Content

Prinzi A, Parker SK, Thurm C, Birkholz M, Sick-Samuels A. Association of endotracheal aspirate culture variability and antibiotic use in mechanically ventilated pediatric patients. *JAMA Netw Open.* 2021;4(12):e2140378. doi:10.1001/jamanetworkopen.2021.40378

eTable 1. Billing Codes

eTable 2. Included Antibiotics

eTable 3. Excluded Antibiotics

eTable 4. Association Between Complex Chronic Conditions and Receiving EAC Testing

This supplemental material has been provided by the authors to give readers additional information about their work.

© 2021 Prinzi A et al. *JAMA Network Open*.

eTable 1. Billing Codes

Study Data Point	Associated Billing Code(s)	Code Description	Relevant keywords	Additional Notes
Mechanical Ventilation	521166<br>521169	Mechanical ventilation<br>Other unspecified ventilator assistance	N/A	CTC daily mechanical ventilation code used rather than ICD-10 code for intubation procedure because the ICD-10 code does not reflect daily ventilation
Lab Test	361110	Aerobic culture	Sources include respiratory tract, sputum, nasopharyngeal, other/unspecified	Hospital charge description keywords: tracheal aspirate, trach, endotracheal, TA, respiratory, nasopharyngeal, sputum

© 2021 Prinzi A et al. *JAMA Network Open*.

eTable 2. Included Antibiotics

**All Antibiotics**	**Anti-Pseudomonas**	**Anti-Staphylococcus**
Amikacin sulfate (parenteral)	Amikacin sulfate (parenteral)	Amoxicillin/clavulanic acid (oral)
Amoxicillin/clavulanic acid (oral)	Aztreonam (parenteral)	Ampicillin/sulbactam (parenteral)
Amoxicillin (oral)	Cefepime (parenteral)	Cefazolin sodium (parenteral)
Ampicillin/sulbactam (parenteral)	Ciprofloxacin (oral, parenteral)	Ceftaroline (parenteral)
Ampicillin (parenteral)	Gentamicin (parenteral)	Clindamycin (parenteral)
Azithromycin (oral, parenteral)	Imipenem (parenteral)	Daptomycin
Aztreonam (parenteral)	Levofloxacin (oral, parenteral)	Linezolid (oral, parenteral)
Cefazolin sodium (parenteral)	Meropenem (parenteral)	Oxacillin (parenteral)
Cefdinir (oral)	Piperacillin/tazobactam (parenteral)	Sulfamethoxazole/trimethoprim (oral, parenteral)
Cefepime (parenteral)	Tobramycin (parenteral)	Vancomycin (parenteral)
Cefotaxime (parenteral)		
Cefoxitin (parenteral)		
Ceftaroline (parenteral)		
Ceftazidime/avibactam (parenteral)		
Ceftazidime (parenteral)		
Ceftolozane/tazobactam (parenteral)		
Ceftriaxone (parenteral)		
Cephalexin (oral)		
Ciprofloxacin (oral, parenteral)		
Clarithromycin (oral)		
Clindamycin (oral)		
Daptomycin		
Doxycycline (parenteral)		
Gentamicin (parenteral)		
Imipenem (parenteral)		
Levofloxacin (oral, parenteral)		
Linezolid (oral, parenteral)		
Meropenem (parenteral)		
Moxifloxacin (oral, parenteral)		
Nafcillin (parenteral)		

© 2021 Prinzi A et al. *JAMA Network Open*.

Oxacillin (parenteral)		
Penicillin G (parenteral)		
Penicillin V (oral)		
Piperacillin/tazobactam (parenteral)		
Polymyxin B sulfate (parenteral)		
Sulfamethoxazole/trimethoprim (oral, parenteral)		
Tobramycin (parenteral)		
Vancomycin (parenteral)		

© 2021 Prinzi A et al. *JAMA Network Open*.

eTable 3. Excluded Antibiotics

Antibiotic Name	Reason for Exclusion
Amikacin, inhaled	Inhaled
Metronidazole	Unlikely to be used clinically
Vancomycin, inhaled	Inhaled, small n (n=5)
Tobramycin, inhaled	Inhaled
Cefixime, oral	Uncommonly used for VAI, small n (n=12)
Tobramycin, inhaled	Inhaled
Cefuroxime, inhaled	Inhaled, small n (n=13)
Cefuroxime, all	Not a typical inpatient drug
Aztreonam, inhaled	Inhaled, small n (n=5)
Tigecycline	Small n (n=4)
Colistin, inhaled	Inhaled, small n (n=10)
Cefuroxime, oral	Not a typical inpatient drug, small n (n=7)
Gentamicin, inhaled	Inhaled, small n (n=6)
Cefotetan	Small n (n=1)
Cefpodoxime	Small n (n=2)
Cefprozil	Small n (n=4)
ceftazidime, inhaled	Inhaled, small n (n=1)
Omadacycline	Small n (n=1)

© 2021 Prinzi A et al. *JAMA Network Open*.

eTable 4. Association Between Complex Chronic Conditions and Receiving EAC Testing

Variable	Total	Ventilated Patients without EAC N (%)	Ventilated Patients with EAC N (%)	P-value
Any Complex Chronic	118,655 (78%)	84,029 (75%)	34,626 (86.5%)	< 0.001
# Complex Chronic Conditions:				
No CCCs	33,477 (22%)	28,052 (25%)	5,425 (13.5%)	
1 CCC	36,649 (24.1%)	30,797 (27.5%)	5,852 (14.6%)	
2 CCCs	22,475 (14.8%)	17,727 (15.8%)	4,748 (11.9%)	
3+ CCCs	59,531 (39.1%)	35,505 (31.7%)	24,026 (60%)	< 0.001
Cardiovascular	56,223 (37%)	39,610 (35.3%)	16,613 (41.5%)	< 0.001
Neurologic and neuromuscular	36,032 (23.7%)	22,039 (19.7%)	13,993 (34.9%)	< 0.001
Respiratory	30,329 (19.9%)	15,290 (13.6%)	15,039 (37.5%)	< 0.001
Renal and urologic	15,557 (10.2%)	10,115 (9%)	5,442 (13.6%)	< 0.001
Gastrointestinal	46,303 (30.4%)	27,640 (24.7%)	18,663 (46.6%)	< 0.001
Hematology and immunodeficiency	8,914 (5.9%)	5,324 (4.8%)	3,590 (9%)	< 0.001
Metabolic	16,218 (10.7%)	9,437 (8.4%)	6,781 (16.9%)	< 0.001
Other congenital or genetic defect	23,752 (15.6%)	15,990 (14.3%)	7,762 (19.4%)	< 0.001
Malignancy	6,512 (4.3%)	4,315 (3.8%)	2,197 (5.5%)	< 0.001
Neonatal	33,900 (22.3%)	24,991 (22.3%)	8,909 (22.2%)	0.826
Technology dependency	55,310 (36.4%)	32,711 (29.2%)	22,599 (56.4%)	< 0.001
Transplantation	2,612 (1.7%)	1,652 (1.5%)	960 (2.4%)	< 0.001

© 2021 Prinzi A et al. *JAMA Network Open*.

© 2021 Prinzi A et al. *JAMA Network Open*.
